# Conformational Space of the Translocation Domain of Botulinum Toxin: Atomistic Modeling and Mesoscopic Description of the Coiled-Coil Helix Bundle

**DOI:** 10.3390/ijms25052481

**Published:** 2024-02-20

**Authors:** Alexandre Delort, Grazia Cottone, Thérèse E. Malliavin, Martin Michael Müller

**Affiliations:** 1 Université de Lorraine, CNRS, LPCT, 57000 Metz, France; 2 Department of Physics and Chemistry-Emilio Segré, University of Palermo, 90128 Palermo, Italy; grazia.cottone@unipa.it; 3 Université de Lorraine, CNRS, LPCT, 54000 Nancy, France

**Keywords:** *Clostridium botulinum*, botulinum toxin, molecular dynamics, residue protonation, mesoscopic *Twister* model, hydrophobicity, water–ethanol solvent

## Abstract

The toxicity of botulinum multi-domain neurotoxins (BoNTs) arises from a sequence of molecular events, in which the translocation of the catalytic domain through the membrane of a neurotransmitter vesicle plays a key role. A recent structural study of the translocation domain of BoNTs suggests that the interaction with the membrane is driven by the transition of an α helical switch towards a β hairpin. Atomistic simulations in conjunction with the mesoscopic *Twister* model are used to investigate the consequences of this proposition for the toxin–membrane interaction. The conformational mobilities of the domain, as well as the effect of the membrane, implicitly examined by comparing water and water–ethanol solvents, lead to the conclusion that the transition of the switch modifies the internal dynamics and the effect of membrane hydrophobicity on the whole protein. The central two α helices, helix 1 and helix 2, forming two coiled-coil motifs, are analyzed using the *Twister* model, in which the initial deformation of the membrane by the protein is caused by the presence of local torques arising from asymmetric positions of hydrophobic residues. Different torque distributions are observed depending on the switch conformations and permit an origin for the mechanism opening the membrane to be proposed.

## 1. Introduction

Botulinum neurotoxins (BoNTs), secreted by *Clostrodium botulinum*, are among the most powerful toxic compounds found in nature, provoking flaccid paralysis of the host [1]. BoNTs are traditionally classified into several serotypes, termed A–G [2] and X [3]. Among them, BoNTs of type A1 are the most used BoNT in medical applications [4].

The toxins are formed by two protein chains connected by one disulfide bridge: the light chain (LC) and the heavy chain (HC). The role of the HC is to prepare the delivery of the catalytic region LC, which is responsible for the toxicity of BoNTs. The HC includes an N-terminal translocation domain (H_*N*_) (≃50 kDa), enabling the delivery of the LC into the cytosol, and a C-terminal domain (H_*C*_) (≃50 kDa), which recognizes specific receptors located at the terminal button of motoneurons. It is generally assumed that the two chains connected by one disulfide bridge are sufficient for producing the toxin’s physiological activity in neuromotors.

Starting from several conformations observed for BoNT/A1 [5] and BoNT/E1 [6], with various protonation schemes depending on physiological pH values of 4.7 and 7, a recent molecular modeling study [7] revealed that the global conformational variability in the toxin mainly depends on the relative displacements of the toxin domains. In addition, the analysis of the relative accessible surfaces according to pH variation pointed to the translocation domain as one of the hotspots for the interaction between the membrane and toxin, in accordance with previous models [1].

**Figure 1:**
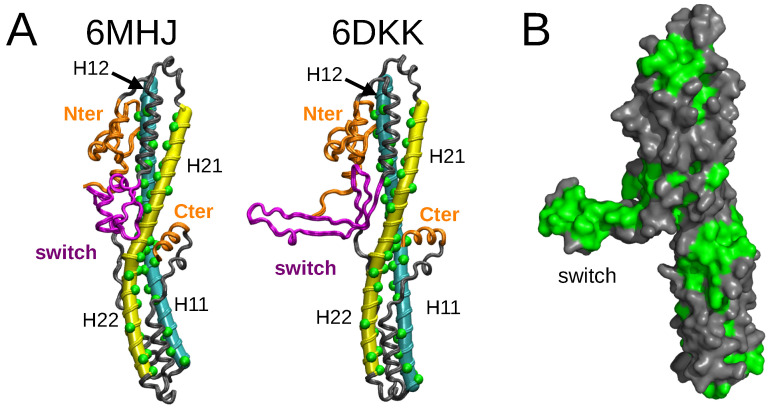
(**A**) X-ray crystallographic structures determined for the translocation domain (PDB entries: 6MHJ determined at pH 8.5 and 6DKK determined at pH 5.1 [8]) are represented as grey ribbons. The tubes generated by Bendix [9] on the helices 1 (cyan) and 2 (yellow) are shown for the two PDB structures. The hydrophobic residues in these helices are represented by green spheres. The limits of the helices are defined as in Table 1. The switch is colored in magenta, and the N-terminal domain and the C-terminal α helix in orange. In helices 1 and 2, the helix halves H11, H12, H21, and H22 are indicated with labels. (**B**) Structure 6DKK represented as a grey surface. The hydrophobic residues GLY, ALA, VAL, LEU, ILE, PRO, PHE, MET, and TRP are colored in green. This image was prepared using pymol [10].

The structures of the *isolated* translocation domain of BoNT/A1 [8] were determined experimentally (Figure 1A) at two pH values, 5.1 and 8.5, which are close to the physiological pH values (acidic and neutral) regulating the function of BoNTs. For the acidic pH value, a dimer of two translocation domains was observed (PDB entry: 6DKK), whereas the other structure (PDB entry: 6MHJ) was monomeric and quite similar to the structure observed within complete toxin structures. In the dimeric structure, the switch domain (residues 620–666 in 6MHJ) undergoes a transition from an α helix bundle to a β strand. The monomer β strands interact with each other, producing a larger β strand which stabilizes the inter-monomer interaction. Lam et al. [8] concluded from their observations that the dimerization is not relevant for BoNT/A1 function in vivo but that the acidification of the inner part of the neurotransmitter vesicle induces a conformational change in the switch, favoring the interaction of the translocation domain with the membrane.

The present work investigates the internal dynamics of the translocation domain in water and in a mixture of water and ethanol with the help of atomistic molecular dynamics (MD) simulations on the hundreds of nanoseconds time scale. Ethanol was chosen as the co-solvent for the following reasons. As the OH group of ethanol is inherently polar, while the CH_3_ group of its short carbon chain exhibits hydrophobic characteristics, ethanol has the ability to engage with proteins through both hydrophilic and hydrophobic interactions. In this context, ethanol has frequently been regarded as a model for investigating the equilibrium between hydrophobic interactions and hydrogen bonds. Last, but most important for the present study, ethanol–water solutions are exploited to mimic the approach of a protein towards the membrane [11], providing access to a diversity of conformations for membrane proteins. A 50/50 water–ethanol ratio is a good compromise to set the ethanol content high enough to force a protein to expose its hydrophobic segments [12] while preventing the aggregation of ethanol molecules, which may impede the weakening of the hydrophobic protein core [13,14,15].

The starting points of the molecular dynamics trajectories are provided by the X-ray crystallographic structures determined by Lam et al. [8] using monomeric conformations. Moreover, we discuss how the protein can deform the membrane after making close contact using a biophysical model. In the past, such models have successfully explained the formation of lipid membrane rafts [16,17], interface-mediated interactions between membrane-bound particles leading to endo-/exocytosis [18], or the formation of membrane channels [19]. To understand how an object containing hydrophobic and hydrophilic parts, such as a biofilament or a protein domain, can interact with a membrane, mesoscopic models are of particular interest. A combination of elasticity theory and geometry helps to describe the various system configurations. Within this framework, two mechanisms have been proposed which can induce the deformation of a membrane due to an object: the *Twister* and the *Darboux torque* mechanisms [20]. Since then, the *Darboux torque* mechanism has been found to explain membrane deformation by FtzsZ [21] and by ESCRT-III [22,23] or membrane fission by dynamin [24]. To the best of our knowledge the *Twister* mechanism has not been used for explaining biological interactions with the membrane and we will explore its applicability in the framework of the BoNT–membrane interaction.

The originality of the work lies not only in the possible emergence of the *Twister* mechanism but also in the implicit modeling of the membrane in the simulation. This choice was motivated by the hypothesis that the inertia of membrane atomistic or coarse-grained systems considerably slows down the events related to the interaction between protein and membrane. The use of a mixed solvent, mimicking the hydrophobic interactions produced by the membrane, allows us to confirm the model of anchoring of the hairpin in the membrane. The final aim of the present work is to obtain information on the protein’s conformational changes at the molecular level, in particular during the interaction with the vesicle membrane, in order to propose mechanistic hypotheses for the translocation of the full toxin through this membrane.

## 2. Results

In this section, we present the results obtained from the protein’s structure and dynamics along the MD trajectories recorded in water and in a 50–50 water–ethanol mixture. We start with an analysis of the internal mobility of the domain, followed by a presentation of the distribution of the solvent molecules. In a final step, the *Twister* model is applied to helices 1 and 2.

In the following, we selected pH values of 4.7 and 7 for acidic and neutral conditions, respectively, although the structures 6MHJ and 6DKK were determined at pH values of 8.5 and 5.1. These values were also those selected to mimic the acidic and neutral conditions inside the vesicles in our previous full-length BoNT MD study [7] as this allows us to directly compare the behaviors of the translocation domain and of the full-length proteins. As in the present study, the pH effect is modeled through different protonation patterns among protein residues, we checked the differences in protein protonation for pH 4.7 vs. 5.1 and pH 7 vs. 8.5 using H++, a method for pK_a_ calculations based on a continuum electrostatic model [25,26] (Table 2). The following protonated residues are predicted at acidic pH, glutamic (GLU) and aspartic (ASP) acids, both protonated on the sidechain carboxyl group. The protonation of one histidine is predicted at pH 8.5. At pH of 4.7, eight residues are detected in 6MHJ, and six in 6DKK. Among them, four residues are located in the switch, one residue in the switch tail, and one residue (GLU-809) in helix 2. The three residues GLU-620, GLU-809, and ASP-848 are protonated in both structures, and for the two pH values 4.7 and 5.1. The protonation at pH 7 or 8.5 is nearly the same for 6DKK and 6MHJ. For acidic pHs, both proteins contain only a few more protonated residues at pH 4.7 compared to pH 5.1.

### 2.1. Internal Mobility of the Translocation Domain

The root-mean-square deviation (RMSD) between atomic coordinates of conformations is utilized to obtain global information on tertiary rearrangements in the protein with time. Atomic root-mean-square fluctuations (RMSFs) are analyzed to investigate local motions in distinct protein regions, while the radius of a cylinder encompassing the protein structure is monitored to complement the global structural results. The combined analysis of RMSD, RMSF, and cylinder radius provides useful insights for tertiary modifications in different conformational and protonation states. The RMSD values were calculated from the atomic coordinates of the initial frame of the simulation, obtained from the PDB structure. The RMSFs were then calculated with respect to the average conformation along the trajectories (Table S1 in the Supplementary Materials).

**Figure 2:**
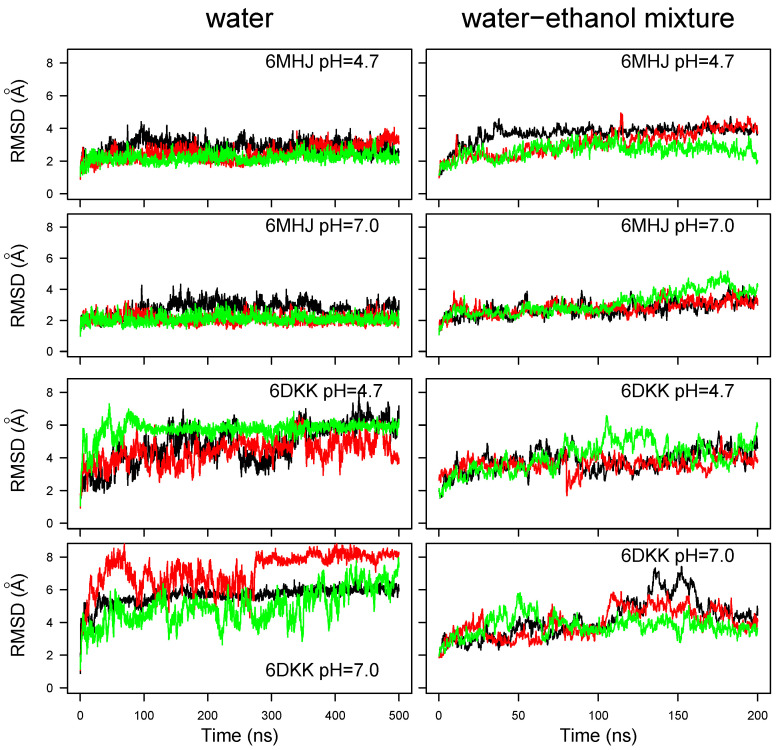
Coordinate root-mean-square deviation (RMSD, Å) along the molecular dynamics (MD) trajectories recorded in water and in 50/50 water–ethanol mixture at the two pH values. Different colors correspond to different replicates of the trajectory.

The coordinate RMSD for the backbone’s heavy atoms along the MD trajectories in water (Figure 2, left panels) display different behaviors for trajectories starting from the momomer (6MHJ) compared to a monomer extracted from the dimer (6DKK). The monomeric structure 6MHJ stays stable with an RMSD plateau around 3 Å, attained after 50 ns of trajectory, whereas the monomer extracted from the dimer structure 6DKK exhibits more heterogeneous RMSD profiles along the trajectories, with plateaus lying between 4 and 8 Å. For a given starting structure and a given solvent, the variation in the protonation does not induce different trends in RMSD variations. However, comparing the global RMSD values for different solvents, the values observed for 6MHJ in water–ethanol increase slightly with respect to the situation in water (Figure 2, upper four panels) whereas the values obtained for 6DKK are reduced (Figure 2, lower four panels). The water–ethanol mixture, thus, seems to produce opposite effects on the two conformations.

These differences observed for the water–ethanol mixture may be put in relation with the physiological effect of pH on the protein, as the two starting conformations were experimentally determined at different pH values. On the one hand, the structure 6DKK was obtained at acidic pH, corresponding to the physiological conditions during the toxin’s interaction with the membrane of the synaptic vesicle. Since the water–ethanol mixture is intended to mimic the hydrophobic effect of the membrane, it is interesting to see that the coordinate RMSD is reduced in the mixture with respect to the observations in water. On the other hand, the structure 6MHJ was observed at neutral pH, corresponding to the physiological conditions of non-interaction with the membrane. Therefore, it is not surprising that the water–ethanol mixture induces a slightly larger drift of coordinates than the water solvent.

The coordinate RMSDs calculated for helices 1 and 2, as well as for the switch, are displayed as boxplots (Figure S1). The helix RMSDs are mostly located in the 1–2 Å range, in agreement with the conservation of the secondary structure of the α helices. Nevertheless, the RMSD values for some 6MHJ replicates of the trajectory are larger than 2 Å in the water–ethanol mixture. In particular, the 6MHJ α helices exhibit RMSD values in the solvent mixture which are slightly larger than the ones observed in water, especially for helix 2. The hydrophobic field produced by the mixed solvent, thus, seems to have a destabilizing effect on the structure 6MHJ. This agrees with the experimental assignment of the structure 6MHJ to the state of the translocation domain in solution [8]. The destabilization propagates also to the switch for one 6MHJ trajectory at pH 7 in the water–ethanol solvent (see the orange box at the bottom right of Figure S1). But, almost no effect of the pH is observed in the helices of 6DKK, either in water or in the ethanol–water mixture. This agrees with the observation of Ref. [27], according to which the secondary structure of the helices does not change with the pH.

In contrast to helices 1 and 2, large RMSD values are observed for the switch in most of the trajectories recorded for 6DKK. These values are larger (in the 5–8 Å range) in water than in the water–ethanol mixture (3–6 Å range). This dispersion recalls the large RMSD drifts observed for 6DKK in water (Figure 2), elucidating that the global conformational drift of the translocation domain is mostly due to the switch region.

**Figure 3:**
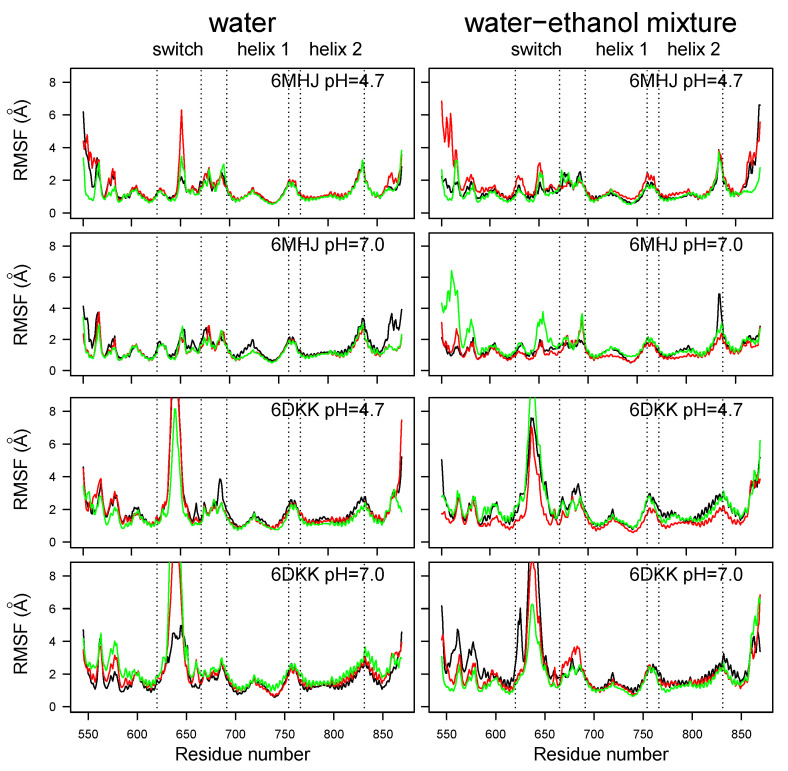
Coordinate root-mean-square fluctuations (RMSFs, Å) calculated on the MD trajectories recorded on the two protein systems. Different colors correspond to different replicates of the trajectory.

The atomic root-mean-square fluctuation (RMSF) profiles (Figure 3) display a large peak located in the region of residues 620–660 corresponding to the switch. This peak is much smaller for 6MHJ than for 6DKK. Several smaller peaks are observed in the N-terminal region, as well as in the loops connecting helices 1 and 2 and the remaining part of the structure. For some trajectories of 6MHJ, a reduction in the pH in water induces the appearance of larger peaks in the switch as well as in the N-terminal region (upper left panel of Figure 3). This gives a hint about the initial transition steps of 6MHJ towards the 6DKK conformation under acidic pH. Large RMSF peaks are also observed for the C-terminal α helix. Looking at the conformational changes in the last frame of the trajectories, the C-terminal α helix undergoes unfolding in the trajectories labeled with ‘u’ (Figure S2), populating conformations similar to those observed in the equivalent region of the X-ray crystallographic structure of BoNT/E1 [6]. In other trajectories, labeled ‘s’ (Figure S2), the C-terminal α helix swings: in the full toxin, this motion would push the receptor-binding domain further away from the translocation domain. This separation of the translocation and the receptor-binding domains has been observed to a more limited extent during the MD simulations recorded on the full toxins [7].

**Figure 4:**
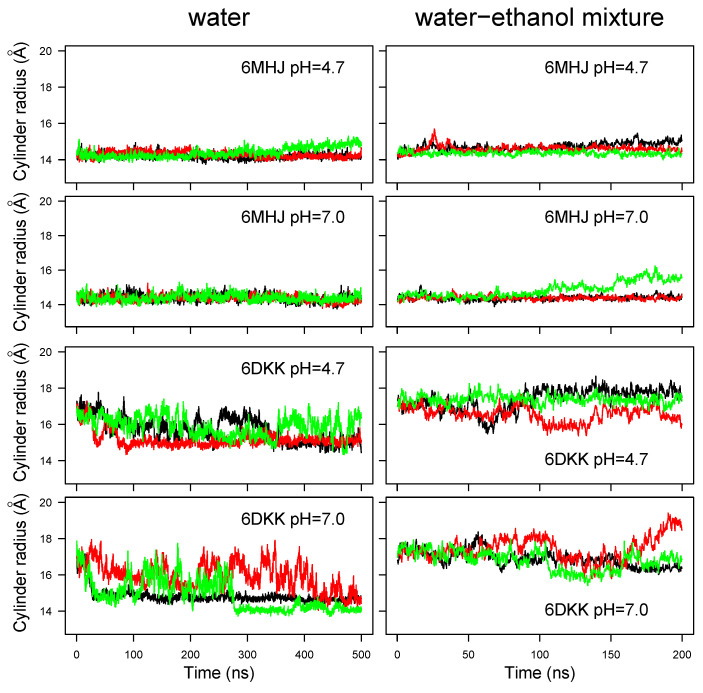
Cylinder radius *r* calculated as $r=\sqrt{2I/m}$, where *I* is the moment of inertia with respect to the axis of the cylinder and *m* is the total molar mass of the Cα atoms. Different colors correspond to different replicates of the trajectory.

As is visible for the conformations displayed in Figure S2, the switch shows various orientations with respect to the remaining part of the domain. In order to obtain a finer picture of the switch positions, the geometry of the protein conformation was modeled as a cylinder, as described in Section 4.3. The time behavior of the cylinder radius (Figure 4) parallels that of the global RMSD (Figure 2). The radius is quite stable around 14 Å for all trajectories recorded on 6MHJ (four top panels of Figure 4). Interestingly, the trajectories display a slight drift at acidic pH (top left panel, green curve), as well as in the water–ethanol mixture, at all pH values. For 6DKK, very heterogeneous profiles are observed for the radius with values in the range of 14.5–19 Å (four bottom panels of Figure 4). Most of the profiles display a decrease in the radius values in water along the trajectory time (bottom left panels). By contrast, the use of the water–ethanol mixture prevents the radius decreasing (bottom right panels). Indeed, the cylinder radius fluctuates around 18 Å, which is the initial radius value arising from the protruding hairpin observed in the PBD entry. This suggests that, in contrast to water, the hairpin keeps protruding from the structure in the water–ethanol mixture, as can be confirmed by visual inspection of the final system snapshots along the trajectories (Figure S2).

Based on all variations observed along the trajectories in water, the strong conformational drift in 6DKK (Figure 2) can be attributed to a variation in the conformation of the switch, due to a tendency to fold onto the remaining part of the translocation domain (Figure S2). Interestingly, the effect is weaker at acidic pH (see conformations in the upper left panel for 6DKK in Figure S2). This folding is due to the large hydrophobic patch present on the switch, which favors the interactions with other hydrophobic patches on the surface of the translocation domain (Figure 1B). The drift agrees with the importance of the dimer interface in stabilizing the protruding conformation of the switch in the 6DKK structure and confirms that the conformation of isolated 6DKK is probably not relevant in water. On the contrary, in the water–ethanol mixture, the hydrophobic field arising from the presence of ethanol allows the extended conformations to be maintained and supports the model of membrane interaction proposed in Ref. [8].

### 2.2. Distribution of Ethanol and Water Molecules around the Translocation Domain

**Figure 5:**
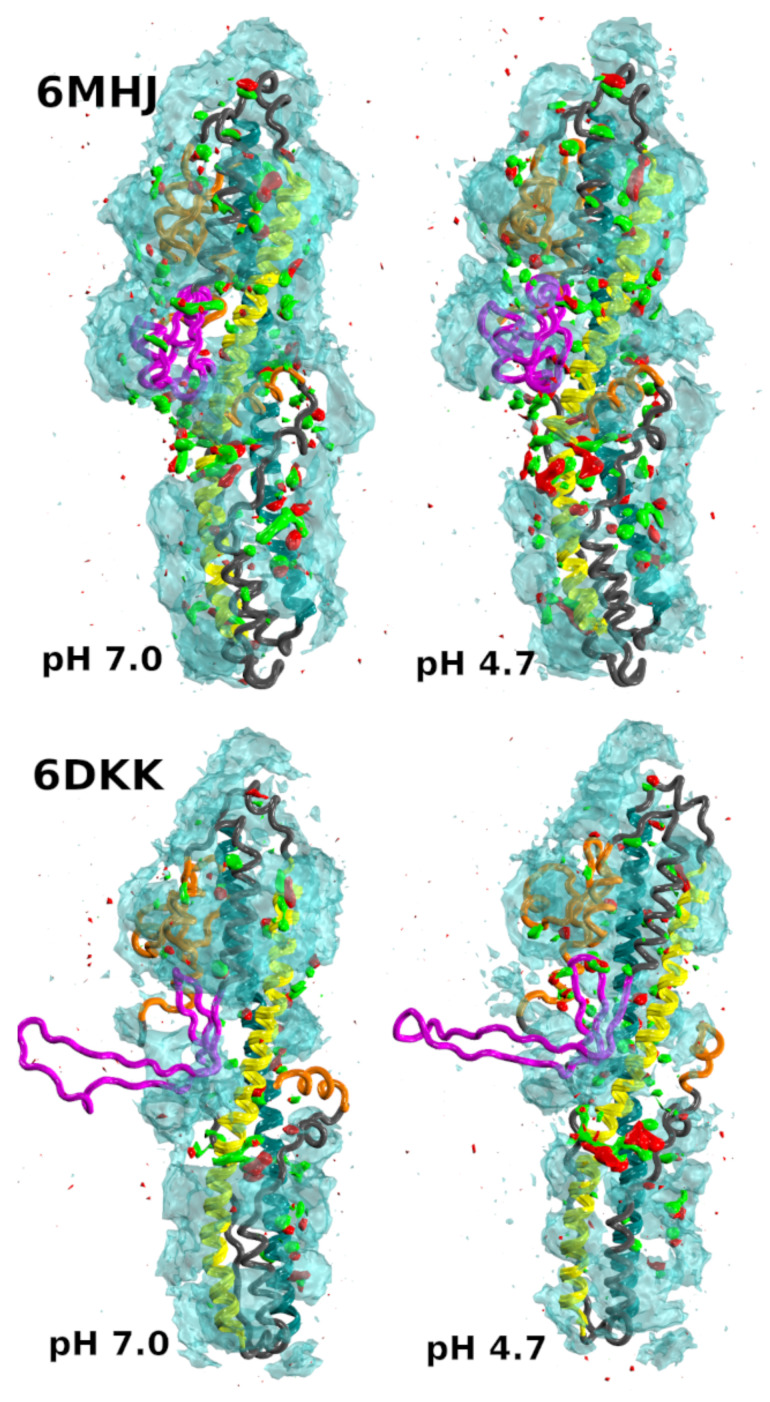
Isosurfaces of the spatial density function of water and ethanol atoms around the protein. Cyan isosurface: water oxygen atoms; red and green: oxygen and methyl carbon atoms, respectively. The isosurfaces are represented at the same isodensity level (0.0115) for both water and ethanol. The protein regions are colored as in Figure 1. Data were collected from the three replicates of the trajectory. This image was prepared using VMD [28].

To obtain insight in the solvent partition close to the protein, the average spatial density functions (SDFs) around the two protein structures in the water–ethanol mixture are calculated and the results are shown in Figure 5.

Overall, the protein is preferentially hydrated. However, ethanol molecules are able to establish interactions with the protein, replacing water. When the solute fluctuates significantly, the resulting SDF of the solvent molecules is averaged to zero. For this reason, the protruding switch conformation in 6DKK displays paradoxically no persistent interaction with ethanol molecules (Figure 5, bottom row). The pH acidification induces a larger number of ethanol molecules to localize in the central region. This agrees with the tendency of the translocation domain to interact with the membrane at the same pH range. Preferential ethanol solvation is, however, more pronounced in the central region of 6MHJ (Figure 5, upper row) than of 6DKK.

In Figure S3, a more detailed comparison of the solvent distributions at the two pH values is shown for 6MHJ only. Preferential ethanol solvation is observed in particular around stretches of hydrophobic residues present in the switch tail and the C-terminal tail (Table 1), and highlighted in a dark gray surface representation. The switch tail, located at the C-terminal of the switch, corresponds to the very hydrophobic primary sequence ILLEFIPEIAIPVLG, whereas the primary sequence STDIPFQLSKYV of the C-terminal tail is less hydrophobic. It is interesting to note that in the protein structure, the switch tail is closer to the protruding switch and possibly to the membrane than the C-terminal tail. The persistent ethanol molecules observed close to the tail regions support a model of protein–membrane interaction in which the tails will be solubilized early during the process.

This solubilization, together with the mobility and unfolding tendency of the C-terminal α helix (Figure S2) and the mobility peaks in several RMSF profiles for the N-terminal domain (Figure 3), suggests that the bundle formed by the two helices 1 and 2 will constitute the remaining piece of the folded structure involved in the interaction between the membrane and the translocation domain. This interaction will be analyzed in the following section with the help of the *Twister* model.

### 2.3. *Twister* Model as a Possible Route for Opening the Vesicle Membrane

To apply a local force or torque distribution on a membrane, and consequently, induce a deformation, forces and torques have to be balanced globally at equilibrium. For a straight filament the *Twister* model suggests that a mismatch between the filament’s internal twist and the hydrophobic regions of the membrane leads to a local torque [20]. The simplest case which cancels the total torque consists of two antiparallel point torques (called the *Twister* in the limit of vanishing distance between the two torques). In the following, we will study whether this model is also relevant for the cases of helices 1 and 2 in the BoNT translocation domain.

For applying the *Twister* model, we need to be sure that the α helix structures are stabilized, at least for the first steps of the interaction with the membrane. This stability is induced by the interaction between the upper and lower halves of the helices observed in the X-ray crystallographic structures (Figure 1A). To support this point, the residues of the regions in contact, 695–713/801–819 and 730–748/766–784, extracted from the PDB entries 6MHJ and 6DKK, were processed using the software CCCP(Coiled-coil Crick Parameterization) [29], available at the web server www.grigoryanlab.org/cccp (accessed on 15 September 2022). This analysis (Table S2) shows that the regions in contact display coiled-coil structural motifs, stabilizing the helix conformations and consequently preserving the predefined asymmetry in the positions of hydrophobic residues. The presence of these coiled-coil motifs will indeed permit the application of local torques during the helix/membrane interaction.

**Figure 6:**
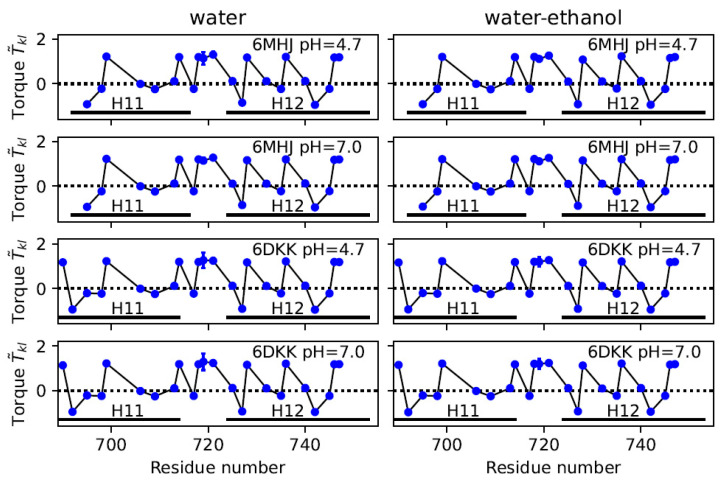
Local torque values ${\tilde{T}}_{kl}$ along helix 1. Values averaged along the first replicate of the trajectory and their standard deviations are plotted as a function of the residue number. Standard deviations are smaller than the size of the data points except for residue ALA-719.

An analysis of the torques ${\tilde{T}}_{kl}$, introduced in Section 4.4, shows that the distribution of the hydrophobic residues along helices 1 and 2 can induce local torques of mostly alternating sign, reminiscent of *Twister*-like interactions (see Figure 6 and Figures S4–S8). The configurations of the helices are stable, which is visible in the coordinates RMSDs being smaller than 2 Å, displayed in Figure S1 and reflected by the tiny standard deviations of ${\tilde{T}}_{kl}$. The only exception is observed for a residue located in the middle of helix 1, ALA-719, which is in close spatial proximity to the switch.

**Figure 7:**
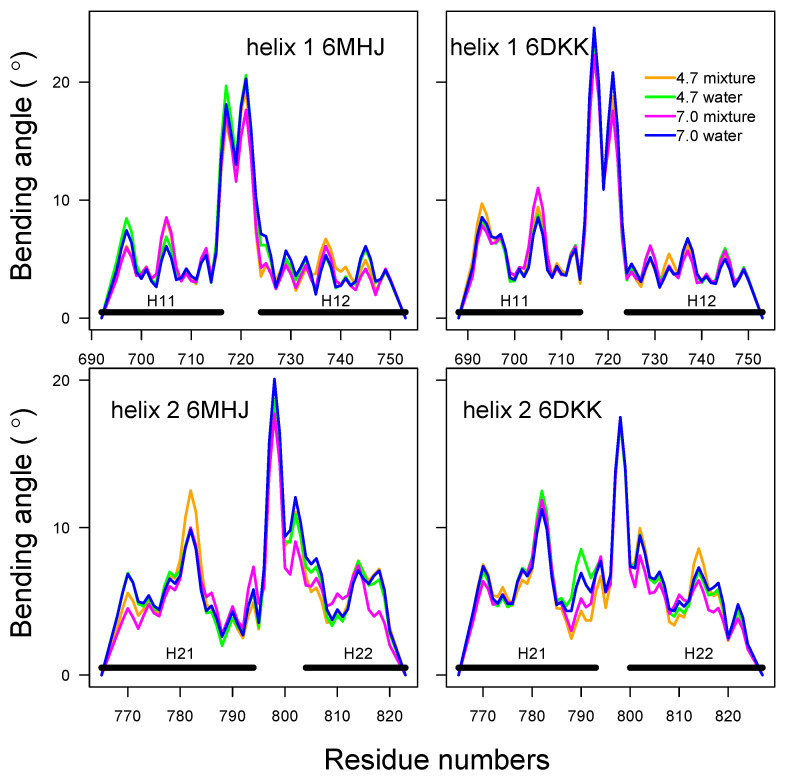
Bending angles of helices 1 and 2. The analysis was performed using the package Bendix [9] along the first replicate of the trajectory.

In order to investigate more deeply the configuration of the helices, we have used the visualization processing Bendix proposed for protein α helices [9]. This processing provides additional information for the helix coarse-graining: first, it shows that a representation as elastic tubes is applicable; and second, it allows the local bending angles along these tubes to be calculated. The bending angles along helices 1 and 2 (Figure 7, Figures S9 and S10) display similar profiles in all conditions. The profiles are almost superimposed for helix 1, whereas small variations with pH and type of solvent are observed for helix 2. A greater sensitivity to the variation of conditions had already been shown for helix 2 in 6MHJ in the subdomain RMSD (Figure S1). The large peaks observed in the middles of the profiles denote a singularity in the definition of the helix axis, implying a kink. This again suggests that the two parts of each helix should be considered separately, in agreement with the previous coiled-coil analysis. We propose, thus, to define four helix parts: H11 and H12 for helix 1, and H21 and H22 for helix 2, with the residue ranges given in Table 1 (see also Figure 1A).

We should first stress that the present study intends to predict the behavior of the protein at the approach towards the membrane [11] based on an in silico analysis of the protein conformations in solution. In this system, helices 1 and 2 display long-range interactions with the other parts of the protein structure, which makes the analysis of the hydrophobic interactions very complex. It is, thus, not simple to draw a conclusion about the *global* equilibrium of the torques, in particular since the membrane is not present explicitly. Nevertheless, we can calculate a total torque acting on each helix part assuming that each part can be modeled as a cylindrical tube. This model is valid in the first approximation since the local bending angle is small within each part (see again Figure 7). We additionally make the (very strong) assumption that the helix bundle at first makes contact with the membrane in the middle between the helix parts, close to the protruding switch. This assumption is supported by the mutagenesis performed by Lam et al. [8]. This implies that the hydrophobic residues at the boundary of each helix part (bold italic in Table S3) are the ones which enter the membrane first, thereby defining the direction of the resulting hydrophobic strip on the helices. The total torque is, thus, obtained by summing up the local torques ${\tilde{T}}_{kl}$ along each region H11, H12, H21, and H22, relative to these residues.

**Figure 8:**
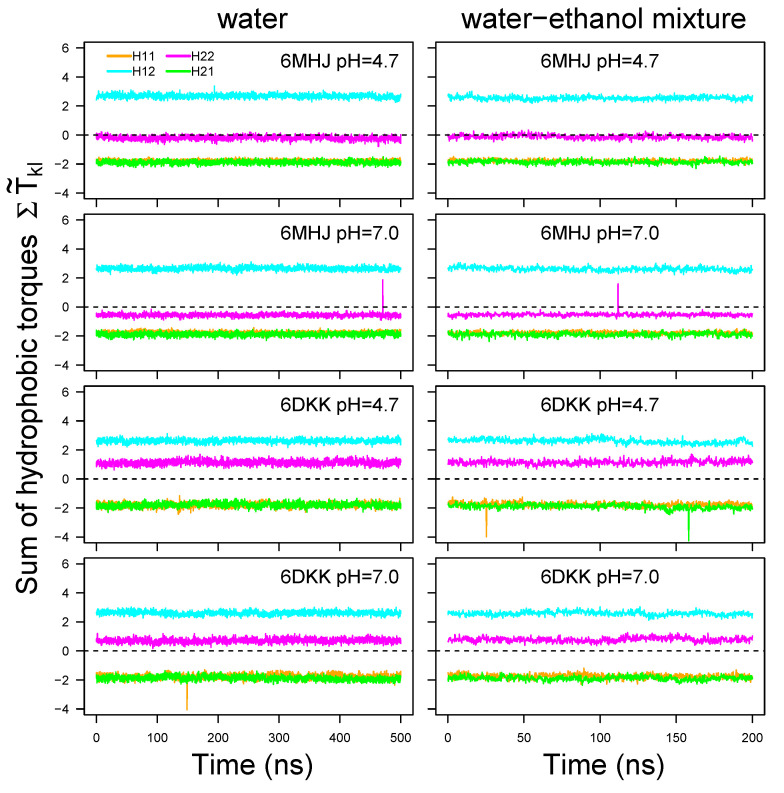
Torque values averaged along the residues 695–713 (H11: orange), 730–748 (H12: cyan), 766–784 (H21: green), and 801–819 (H22: magenta) and plotted along the trajectory time. H12 and H21 are interacting through a coiled-coil motif, as well as H11 and H22. For pH 7, these values were, respectively, calculated on the following sets of hydrophobic residues: I695, A698, L699, W706, V709, and I713 for H11, M732, A735, L736, A740, A742, A745, I746, and I747 for H12, I766, L769, L773, I777, A780, M781, I782, and I784 for H21, I801, P802, G804, V805, L808, F811, A813, L815, A818, and L819 for H22. For pH 4.7, the protonated residue E809 should be added to the list of H22. Note that the curves of H11 and H21 superimpose on each other. Dashed lines indicate the zero line.

The resulting total torques fluctuate only marginally around a constant value along the trajectory time (Figure 8, Figures S11 and S12). The type of solvent does not induce much variation in the torque values. Changing the protonation scheme slightly shifts the value for H22, since one additional residue (GLU-809) is protonated at pH = 4.7. The largest difference is observed between the 6MHJ and 6DKK structures: the value for H22 is negative and close to zero for 6MHJ and positive for 6DKK. Due to the existence of the coiled-coil motifs we assume a direct interaction of the region H11 with H22 and of the region H12 with H21. For the local torques to act on the membrane, the global torque has to vanish. This is approximately the case for the conformation 6DKK at pH = 4.7 for both coiled-coil motifs. Interestingly, this is the structure obtained at conditions (acidic pH and protruding switch) pointed out [8] as characteristic for the interaction with the membrane.

The opposite torque values suggest a mechanism in which the helix halves in direct interaction *via* the coiled-coil motifs (H11/H22 and H12/H21) undergo opposite torques. These global torques could induce additional rotations of each helix with respect to the other, inducing the hydrophobic residues involved in the coiled-coil interactions to insert between the lipid tails of the membrane.

In the structures of the translocation domain studied here, helices 1 and 2 are surrounded by the N-terminal domain, the switch domain, the C-terminal α helix and the tails connecting the switch and helix 1 and connecting helix 2 to the C-terminal helix (Table 1). From the previous analyses (Figure S2), the C-terminal helix displays a tendency to unfold. In addition, sites of ethanol binding are observed on the tails (Figure S3). Moreover, Lam et al. proposed that the β hairpin switch anchors the protein to the membrane [8]. Putting all that together, it is quite probable that, at a certain stage of the membrane interaction, helices 1 and 2 correspond to the last folded part of the translocation domain. At that stage, the torque profile plays, thus, an essential role for predicting the conformational transition of the two coupled tubes in the membrane.

## 3. Discussion

In this work, the conformational dynamics of the BoNT translocation domain was investigated by molecular dynamics simulations. Two different experimental structures were employed as starting points of the simulations, performed at acidic and neutral pH values. The protein–membrane interaction was implicitly taken into account by using a water–ethanol mixture to mimic a hydrophobic environment. The ethanol displays a polarity of 0.6 on a scale where water is at 1, and hexane at 0 [30]. Ethanol is formed of a polar group (OH) and of a hydrophobic tail (ethane), displaying some similarity with the lipid molecules, formed of a polar head and a tail. In the view of the authors, the water–ethanol mixture provides a numerical trick for testing the hydrophobic interactions of the protein as the ethanol molecules are smaller than the lipids and can interact more freely with the protein surface. The reliability of water–ethanol for mimicking the effect of the membrane is supported by the behavior of the switch in the water–ethanol mixture. In contrast to the observations in aqueous solution, the switch was indeed shown to keep its protruding position in mixed solvent. This also confirms that the protruding conformation is probably the one selected in establishing the first interaction with the membrane.

The present work may also be valuable in the frame of enhanced sampling approaches [31,32], as a key step in these methods is the choice of collective variables, defining the coordinate of reaction for the exploration of the conformational space of biological systems. In the case of soluble proteins undergoing translocation across the membrane, this choice is very delicate as the distribution of hydrophobic residues on the protein surface cannot give an indication, in contrast to the transmembrane proteins. The procedure described in this work could provide information for the choice of such collective variables. One possible choice would be the angles between the axes of H11 and H12 and between the axes of H21 and H22 to drive rotation around the pivot, and rotation of each of these helix moities around their own axis to favor the exposure of hydrophobic residues. It is worth noting that the use of a model issued from the soft matter field allowed new light to be shone on this problem. We took advantage of the mesoscopic model of filament interactions [20] to mimic the cooperative effect of the numerous lipids present in an ordered way in the membrane. Indeed, the torque produced by the asymmetry of the positions of hydrophobic residues in the protein can only be active in the presence of a formed membrane, stabilized by the cooperativity of the interactions between lipids.

Based on the conformations explored at the atomistic level, the concomitant interaction between the coiled-coil helices and the membrane was investigated in the frame of the mesoscopic *Twister* model, which permits to calculate the torques underlying the membrane–protein interaction, required for membrane opening. The introduction of this model is the most innovative point of the work presented here. Its novelty lies in the connection established between conformations at atomic resolution and the mesoscopic scale. A possible extension would be to use an adapted scale of hydrophobicity for the amino acid residues. Various scales could be exploited in that respect [33,34]. Nevertheless, the present state of the model only allows for a qualitative description of protein–membrane interaction. A description at atomic resolution of this interaction would require the development of a related energy function. Moreover, while a lipid–protein complex relies on relatively localized interactions, in principle the ethanol–protein interaction takes place *via* diffuse contacts. However, the analysis of the ethanol distribution around the structure 6DKK at pH 4.7 revealed some strong preferred localization of ethanol methyl groups close to the center of the translocation domain (large green spots in Figure 5, lower right panel). Knowing that the switch, located at the center of the domain as well, also has some preference for insertion into the membrane, these two pieces of information permit a local pivot point to be inferred for applying the torques arising from the asymmetry in the positions of the hydrophobic residues.

The implication of coiled-coil motifs in this study is not surprising, since these motifs are known to play a role in many other structures interacting with membranes, such as in the SNARE complex [35]. In addition, successful examples in the design of membrane systems used coiled-coil motifs as a starting point. For example, rational de novo design of channels was possible based on αHB coiled-coil peptides [36]. Moreover, a liposomal fusion model system was developed [37] using a set of hetero-dimeric coiled-coil peptide pairs.

The *Twister* analysis of the torques along the helix tubes permits a model to be proposed in which local torques induce membrane deformations. The resulting global opposite torques within the coiled-coil motifs of helices 1 and 2 additionally imply a rotation of the motif H11 with respect to H22 and of H12 with H21. The middle regions of helices 1 and 2 were not included in the torque analysis, since they were not part of a coiled-coil motif. As these regions are close to the β hairpin switch, their unfolding could, however, permit a reorganization of the four helix motifs, potentially inducing the formation of a pore in the membrane. Interestingly, the beltless translocation domain of botulinum toxin A was shown to be able to act as a conductive channel [38], reminiscent of the design experiments on coiled-coil motifs [36,37].

The analysis of the trajectories at various pH values and in polar and more hydrophobic solvents allows us to propose hypotheses on the interactions between the *whole* botulinum toxin (BoNT) and the membrane of neurotransmitter vesicles. Firstly, upon acidification the translocation domain adopts a conformation with a protruding hairpin to initiate the interaction with the membrane. Secondly, the unfolding of the C-terminal α helix agrees with the observations made on the BoNT/E1 structure [6]: the C-terminal α helix (residues 821–845 in BoNT/E1) of the translocation domain unfolds and displays loop conformations. The translocation domain, thus, behaves relatively independently from the remaining BoNT structure, in agreement with the modularity previously observed between various regions of BoNTs [7]. The most stable sub-structures of the translocation domain are the coiled-coil motifs formed between helices 1 and 2; these motifs apply torques opening the membrane, possibly inducing pore formation. Several models of the subsequent passage of the catalytic domain of BoNTs have been proposed [39]. An investigation of the actual translocation mechanism is out of the scope of the present study.

In summary, this study represents a first step in coupling the mesoscopic *Twister* model to an atomistic approach with predictive power for protein–membrane interactions. The concomitant use of the two approaches allowed a hierarchy to be defined in the interactions between the translocation domain and the membrane. This hierarchy proposed a division of the protein structure in regions undergoing unfolding or separation from the protein core, and the bundle of helices 1 and 2 as the main player involved in membrane insertion. Our predictions are of particular interest in the case of soluble proteins displaying conformational changes during their insertion in the membrane. As structural biology techniques often have difficulties in capturing these changes due to the dynamics of the process, the model described here might provide low-resolution information which can be probed experimentally or combined with high-resolution structures.

## 4. Materials and Methods

### 4.1. Preparation of the Starting Conformations of Toxins

The monomeric (6MHJ) and dimeric (6DKK) structures of the translocation domain [8] were downloaded from the Protein Data Bank www.rcsb.org (accessed on 13 June 2022). In 6MHJ, the residue F658 was modified into E658 in order to fit to the WT sequence of BoNT/A1. In 6DKK, chain B was extracted as a monomer as it contained the smallest number of missing residues at the C-terminal extremity of the protein. As already mentioned in the introduction, a 6DKK monomer was studied by molecular dynamics as the dimer was shown [8] to be induced by crystallogenesis.

The studied systems were named (Table S1) using the PDB entry name (6MHJ/6DKK), the pH for which the protonation was defined (47 or 70), and the solvent name (W for TIP3P and E for water–ethanol). The names of these systems are also the names of the corresponding trajectories.

### 4.2. Molecular Dynamics Simulations

For each previously described system, the protein was embedded in a water box (Table S1) and counterions were added to neutralize the net system charge. The total number of atoms varies between 97,483 and 189,355 (Table S1). All molecular dynamics (MD) simulations were performed using NAMD 2.13 [40] and NAMD 3.0 [41] depending on the type of machine architecture. The CHARMM36 force field [42] for the protein and the TIP3P model for the water molecules [43] were used. A cutoff of 12 Å and a switching distance of 10 Å were employed for non-bonded interactions, while long-range electrostatic interactions were calculated with the Particle Mesh Ewald (PME) method [44]. Before starting each MD trajectory, the system was minimized for 20,000 steps, then heated up gradually from 0 to 310 K in 31,000 integration steps. Finally, the system was equilibrated for 500,000 steps in the NVT ensemble at 310 K, yielding a trajectory of 1 ns. In these first three stages, all carbon α (Cα) atoms were kept fixed. Simulations were then performed in the NPT ensemble, (P=1bar, T=310K), with all atoms free to move. During the equilibration and production trajectories, the non-iterative Settle algorithm [45,46] was used to keep all covalent bonds involving hydrogen atoms rigid, enabling a time step of 2 fs. The atomic coordinates were saved every 20 ps. A total of 500 ns of production was recorded and each trajectory was triplicated for a cumulative trajectory duration of 1.5 μs.

The 50/50 mixed water–ethanol systems were prepared in the following way. The software PACKMOL (version 20.3.5, Martinez Molecular Modeling Group (Campinas, SP, Brazil)) [47] was run to generate boxes of water and ethanol molecules, with the same number of each molecule type (water–ethanol box in Table S1). This box was then minimized, thermalized and equilibrated for 1 ns using the same protocol as the one described above for the protein in water. The final frame was then superimposed to the protein’s initial conformations solvated in a water box, and each water or ethanol molecule located at less than 2.4 Å from the protein atoms was discarded from the system. The NAMD coordinate and topology input files were generated and the minimization, thermalization, equilibration and production protocol described above was used to simulate the protein–ethanol–water systems. 200 ns of production were recorded and each trajectory was triplicated for a cumulative trajectory duration of 0.6 μs. For the trajectories recorded on 6DKK, roto-translational motions of the protein were harmonically restrained throughout the simulation (with a scaled force constant of 30 kcal/mol) using the colvar commands [48] to avoid any rigid-body motion of the protein but leaving the internal dynamics unaltered.

### 4.3. Analysis of the Atomistic Trajectories

The analysis of protein conformations sampled along MD trajectories was realized using ccptraj [49] and the python package MDAnalysis [50,51]. The bending angles of helices 1 and 2 (Figure 1A) in H_*N*_ were determined using the VMD plugin Bendix [9] every 10 frames of the trajectory.

Due to the elongated structure of the translocation domain, the whole shape of the domain was analyzed using a model based on a cylinder. Using the MDAnalysis library [51], the moments of inertia and principal axes were calculated from the positions of the Cα atoms every 10 frames of the trajectory. With the help of the moment of inertia and the principal axes of the translocation domain, the average radius *r* of a cylinder superposed to the protein was calculated as $r=\sqrt{2I/m}$, where *I* is the moment of inertia with respect to the axis of the cylinder and *m* is the total molar mass of the Cα atoms.

To analyze the solvent partition in the protein domain, the spatial density function (SDF) distribution of the solvent molecules around the protein was calculated using the VolMap tool provided by VMD [28]. After translational and rotational fitting of the system to a reference conformation of the protein (the one in the initial frame), the positions of selected solvent atoms (the oxygen atom in water, the oxygen atom of the hydroxyl moiety, and the carbon atom of the methyl group in the ethanol) were mapped onto a cubic grid with a grid spacing of 1 Å centered on the protein. The grid densities were then averaged over the number of analyzed frames.

### 4.4. Coarse-Grained Modeling of Helices 1 and 2

Helices 1 and 2 (Figure 1A) were described in a coarse-grained manner: we assumed that they were elastic cylindrical tubes. In that framework, the axes of helix 1 and helix 2 were defined using the method originally developed by Kahn [52]. The starting and last protein residues of the tubes were fixed using the helix limits given in Table 1. The hydrophobic residues considered in a given α helix were selected in the following way: the residues GLY, ALA, VAL, LEU, ILE, PRO, PHE, MET, and TRP, classically considered as hydrophobic, supplemented at pH 4.7 with the protonated residue GLU-809. Each tube could interact with the membrane *via* the hydrophobic attraction between the protein residues and the lipid tails of the membrane bilayer. To understand this interaction, we had to consider the orientations of the residues with respect to each other.

**Figure 9:**
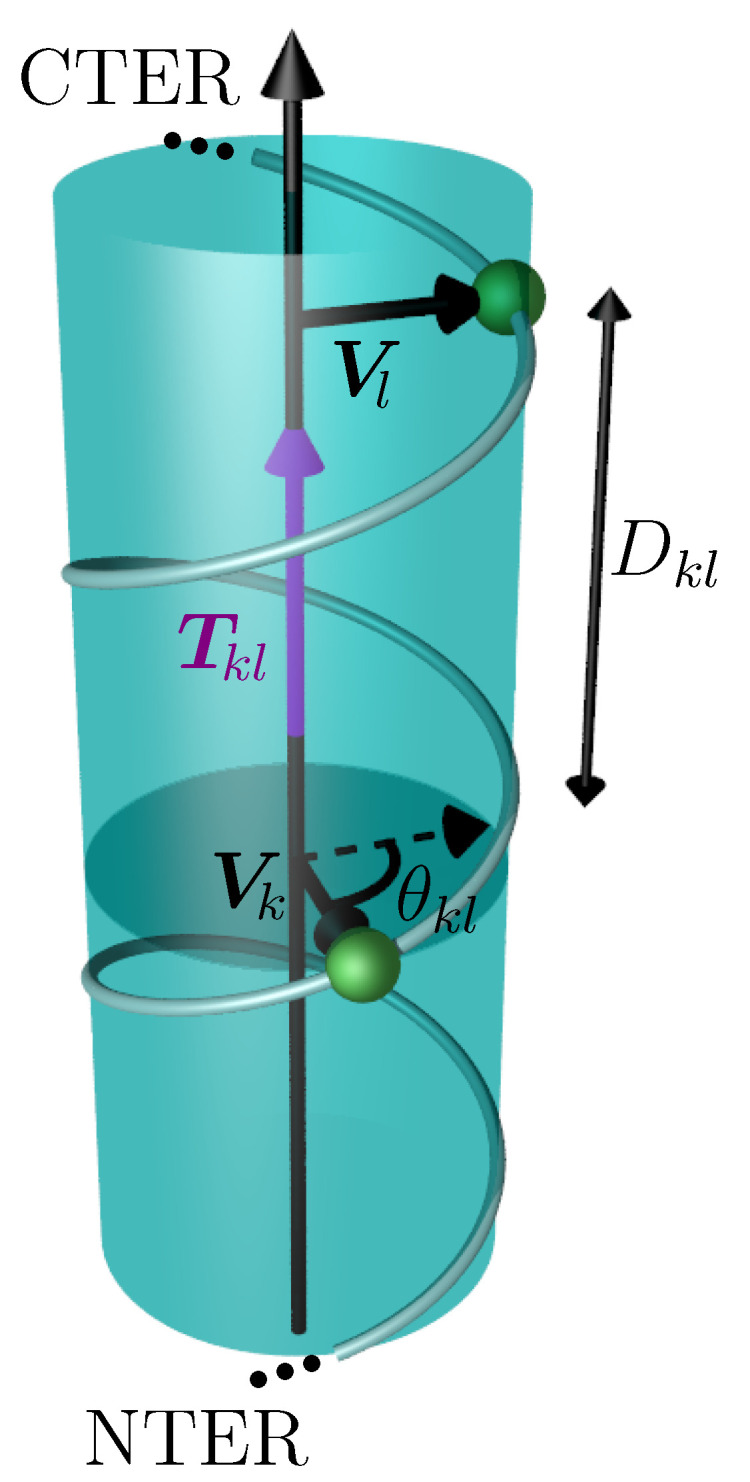
Geometry of the coarse-grained α helix describing the calculation of the torque $T_{kl}$ (in purple). The α helix is represented by a transparent cylinder, the helix axis is parallel to the torque. The two vectors $V_{k}$ and $V_{l}$ are perpendicular to the helix axis and connect the axis to the Cα atoms of the two consecutive hydrophobic residues *k* and *l* (in green). The projection of $V_{l}$ next to $V_{k}$ (dashed vector) is drawn in order to illustrate the definition of $\theta_{kl}$.

Using the relative positions of hydrophobic residues *k* of the considered α helix (Table S3), a set of vectors was determined which are normal to the axis. Each vector $V_{k}$ connects the axis to the Cα atom of residue *k* (see Figure 9). If all of these vectors were parallel, the hydrophobic residues would align on a straight line, and the helix would be amphipathic and could insert itself into the membrane without undergoing an elastic deformation. When the angle $\theta_{kl}$ between $V_{k}$ and $V_{l}$ of two consecutive hydrophobic residues *k* and *l* is non-zero, the tube has to twist to align the two residues. The torque $T_{kl}$ resulting from the non-alignment of the residue is proportional to the angle $\theta_{kl}$ in the lowest order and anti-proportional to the distance $D_{kl}$ between the points $P_{k}$ and $P_{l}$. For a cylindrical tube, $T_{kl}$ is given by [53,54,55]

(1) $$T_{kl}=JG\frac{\theta_{kl}}{D_{kl}}\;,$$

where $J=\frac{\pi }{2}R^{4}$ is the polar moment of inertia of the tube’s cylindrical cross-section with radius *R* and *G* is the shear modulus. In the following, we will use the scaled torque ${\tilde{T}}_{kl}:=\frac{T_{kl}}{JG}$. For proteins, due to the variability in their structures, only sparse experimental information is available for the value of GJ. Indeed, only few cases have been studied in detail: values for a large protein complex (IgG/G protein) are of the order of 45,000 $\mathrm{pN}{\mathrm{nm}}^{2}$, whereas a value of only 400 $\mathrm{pN}{\mathrm{nm}}^{2}$ was found for the flexible DNA double helix [54,55]. From the point of view of sizes and internal mobility the BoNT helices 1 and 2 are in between these two cases. We can, thus, expect values between the numerical values quoted above.

The torque vectors are all parallel to the axis. A positive sign of ${\tilde{T}}_{kl}$ indicates that the vector is aligned from the N- to the C-terminal extremities of the helix sequence, whereas it is aligned in the opposite direction when the sign is negative. A python script using the library MDAnalysis [51] was specifically developed for the calculation of the torques.

## Figures and Tables

**Table 1:** Definition of protein domains in the translocation domain H_*N*_. Differences between the two structures are highlighted in bold face. The regions H11 and H12 correspond to parts of helix 1 in interaction with the regions H21 and H22 of helix 2 through the coiled-coil motifs (see Section 2.3).

Domain	Residues Range 6MHJ	Residues Range 6DKK
N-terminal	547–596	547–596
Switch	620–666	620–666
Switch tail	663–677	663–677
Helix 1	**692**–753	**688**–753
H11	**692**–**716**	**688**–**714**
H12	724–753	724–753
Helix 2	765–**823**	765–**827**
H21	765–**794**	765–**793**
H22	**804**–**823**	**800**–**827**
C-terminal tail	846–857	846–857
C-terminal α helix	860–**871**	860–**870**

**Table 2:** List of protonated residues according to the studied system. These residues correspond to glutamate protonated on sidechain carboxyl (GLU/E), to aspartic acid protonated on sidechain carboxyl (ASP/D), and to one histidine protonated on Hϵ2. The residues protonated in both structures at a given pH value are written in bold, and the corresponding regions defined for each structure in Table 1 are given in parentheses.

6MHJ (pH values)			
4.7	5.1	7.0	8.5
GLU-558 (N-terminal),	**GLU-620** (switch),	-	HIS-561 (N-terminal)
**GLU-617**,	**GLU-809** (helix 2),		
**GLU-620** (switch),	ASP-625 (switch),		
**GLU-809** (helix 2),	**ASP-848** (C-terminal tail)		
ASP-625 (switch),			
ASP-629 (switch),			
GLU-758,			
**ASP-848** (C-terminal tail)			
**6DKK (pH values)**			
4.7	5.1	7.0	8.5
**GLU-617**,	GLU-617,	-	-
**GLU-620** (switch),	**GLU-620** (switch),		
GLU-670 (switch tail),	**GLU-809** (helix 2),		
**GLU-809** (helix 2),	**ASP-848** (C-terminal tail)		
ASP-616 (switch),			
**ASP-848** (C-terminal tail)			

## Data Availability

The datasets supporting the conclusions of this article are available online: https://doi.org/10.5281/zenodo.10680007.

## Supplementary Materials

The following supporting information can be downloaded at: https://www.mdpi.com/article/10.3390/ijms25052481/s1.
