# Supplementary material for: Conformational Space of the Translocation Domain of Botulinum Toxin: Atomistic Modeling and Mesoscopic Description of the Coiled-Coil Helix Bundle
Source: Int J Mol Sci. 2024 Feb 20;25(5):2481. doi: 10.3390/ijms25052481 (PMC10931374; doi:10.3390/ijms25052481)
Supplement: Supplementary file 1 [file ijms-25-02481-s001.zip › ijms-2814367-supplementary.pdf]

Supplementary Information: Conformational Space of the Translocation Domain of Botulinum Toxin: Atomistic Modeling and Mesoscopic Description of the Coiled-Coil Helix Bundle

Alexandre Delort (1), Grazia Cottone (3), Thérèse E. Malliavin (2), Martin Michael Müller (1)

(1) Université de Lorraine, CNRS, LPCT, 57000 Metz, France

(2) Université de Lorraine, CNRS, LPCT, 54000 Nancy, France

(3) Department of Physics and Chemistry-Emilio Segré, University of Palermo, Palermo, Italy

Corresponding authors:

Thérèse E. Malliavin, Therese.Malliavin@univ-lorraine.fr

Martin Michael Müller, Martin-Michael.Mueller@univ-lorraine.fr

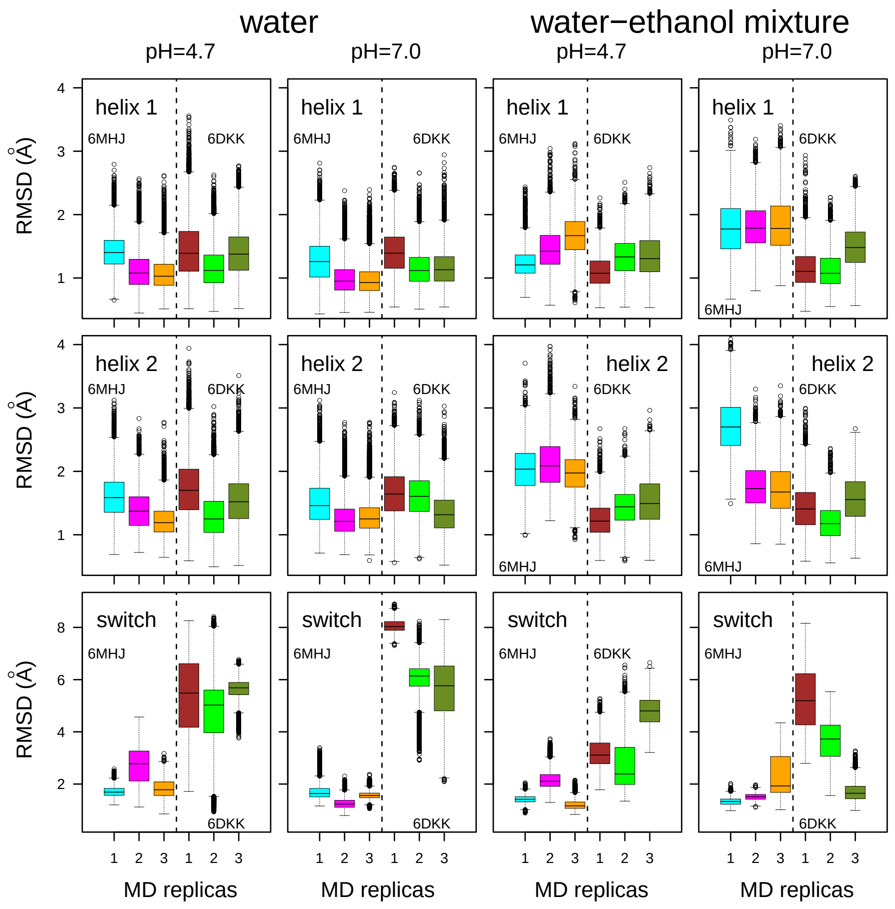

Figure S1: Distributions of coordinate RMSD (Å) for specific domains of the protein.

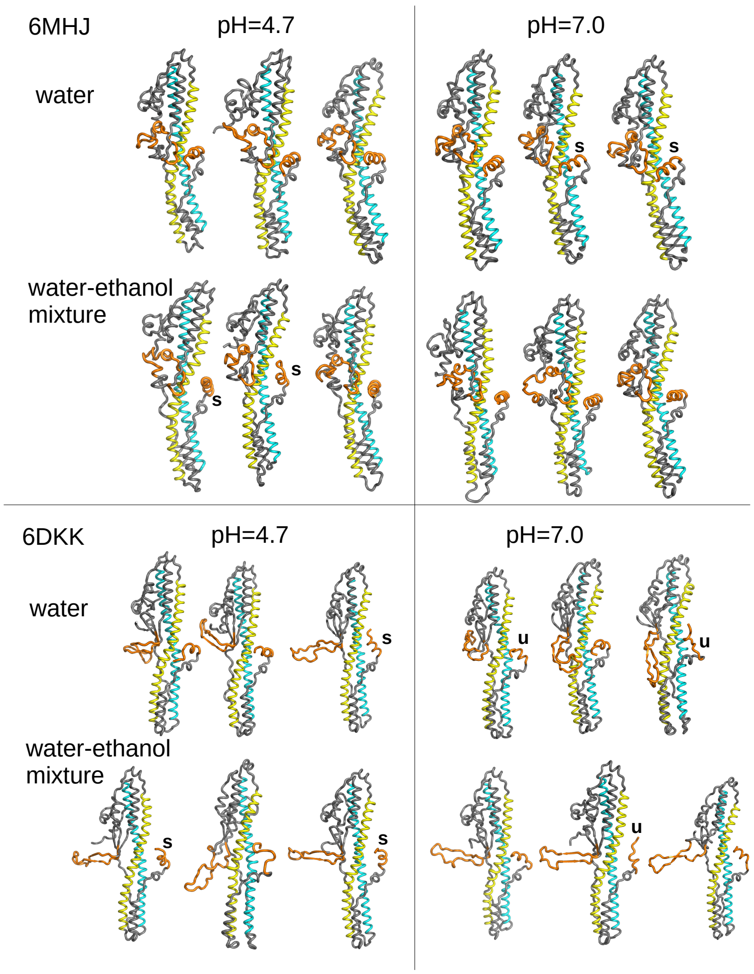

Figure S2: Final conformations of the replicates of the three trajectories in each investigated system. The protein is represented in ribbon; the switch region and the C terminal α helix are colored in orange and the helices 1 and 2 in cyan and yellow, respectively. Examples of unfolded C terminal α helices are labeled with 'u', and examples of different orientations of the C terminal α helices are labeled with 's'.

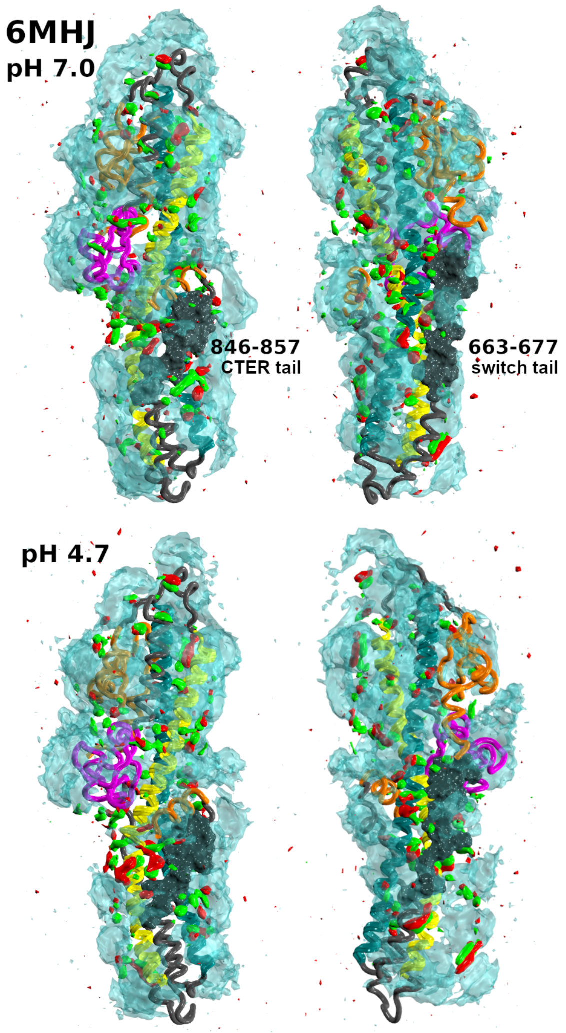

Figure S3: Isosurfaces of the Spatial Density Function of water and ethanol around the protein, represented as in Figure 6 in the main text. The isodensity value is 0.0115 for all atomic species. The protein regions are colored as in Figure 1. The residue stretches 663-677 and 846-857 (switch and CTER tails, respectively) are highlighted in dark grey surface representation. Data were collected from the replicates of the three trajectories. This image was prepared using VMD [1].

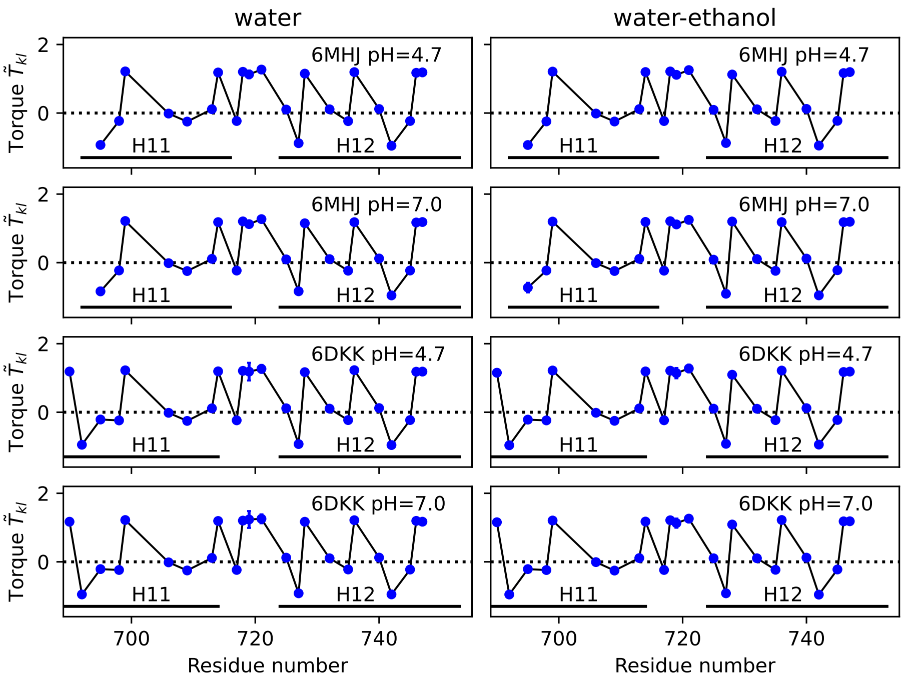

Figure S4: Local torque values $\tilde{T}_{kl}$ along the helix 1. Values averaged along the second replicate of trajectories and their standard deviations are plotted as a function of the residue number. Standard deviations are smaller than the size of the data points except for residue ALA-719.

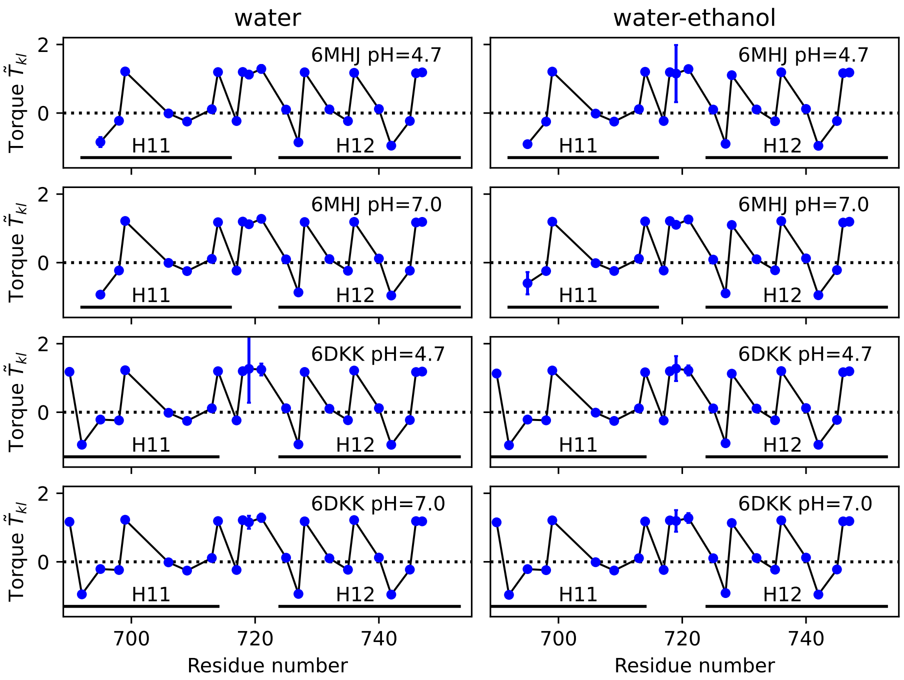

Figure S5: Local torque values $\tilde{T}_{kl}$ along the helix 1. Values averaged along the third replicate of trajectories and their standard deviations are plotted as a function of the residue number. Standard deviations are smaller than the size of the data points except for residue ALA-719.

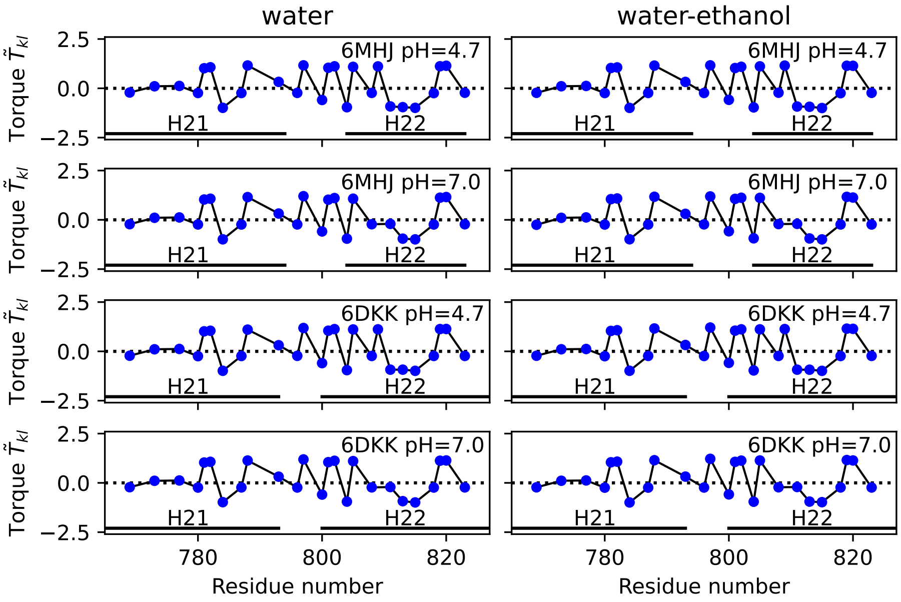

Figure S6: Local torque values $\tilde{T}_{kl}$ along the helix 2. Values averaged along the first replicate of trajectories and their standard deviations are plotted as a function of the residue number. Standard deviations are smaller than the size of the data points.

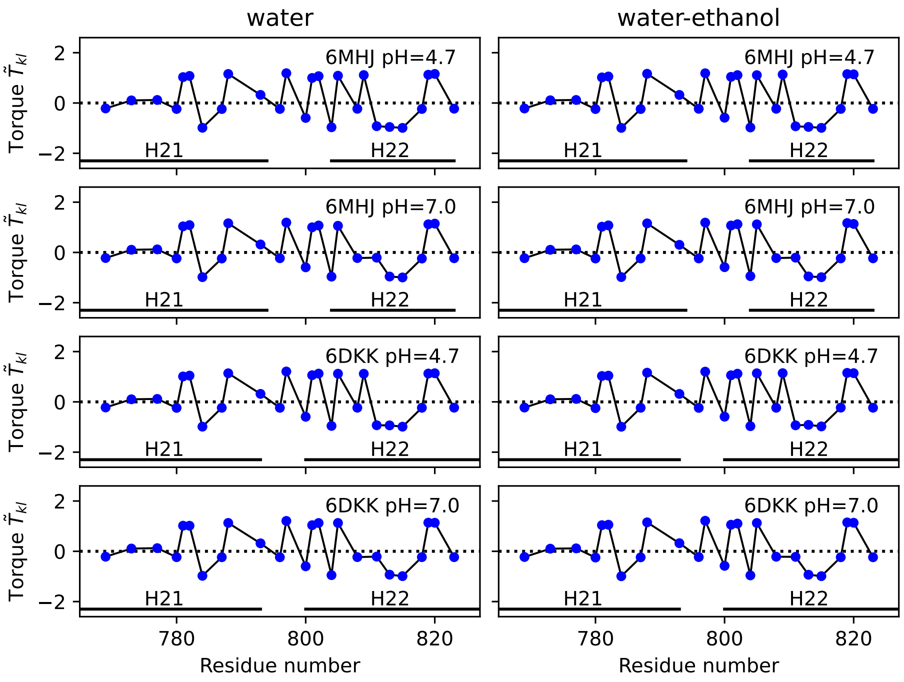

Figure S7: Local torque values $\tilde{T}_{kl}$ along the helix 2. Values averaged along the second replicate of trajectories and their standard deviations are plotted as a function of the residue number. Standard deviations are smaller than the size of the data points.

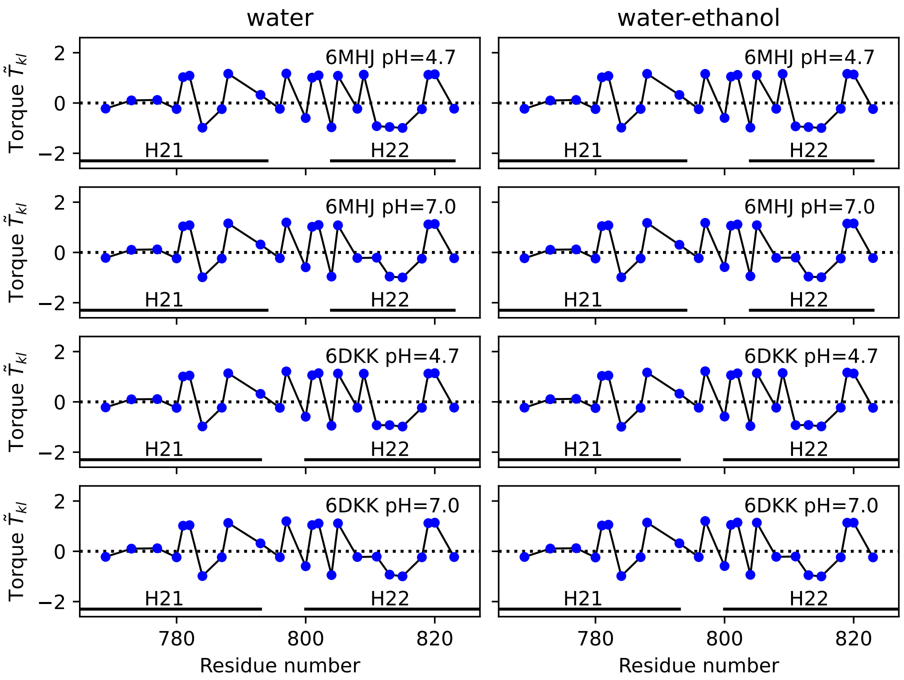

Figure S8: Local torque values $\tilde{T}_{kl}$ along the helix 2. Values averaged along the third replicate of trajectories and their standard deviations are plotted as a function of the residue number. Standard deviations are smaller than the size of the data points.

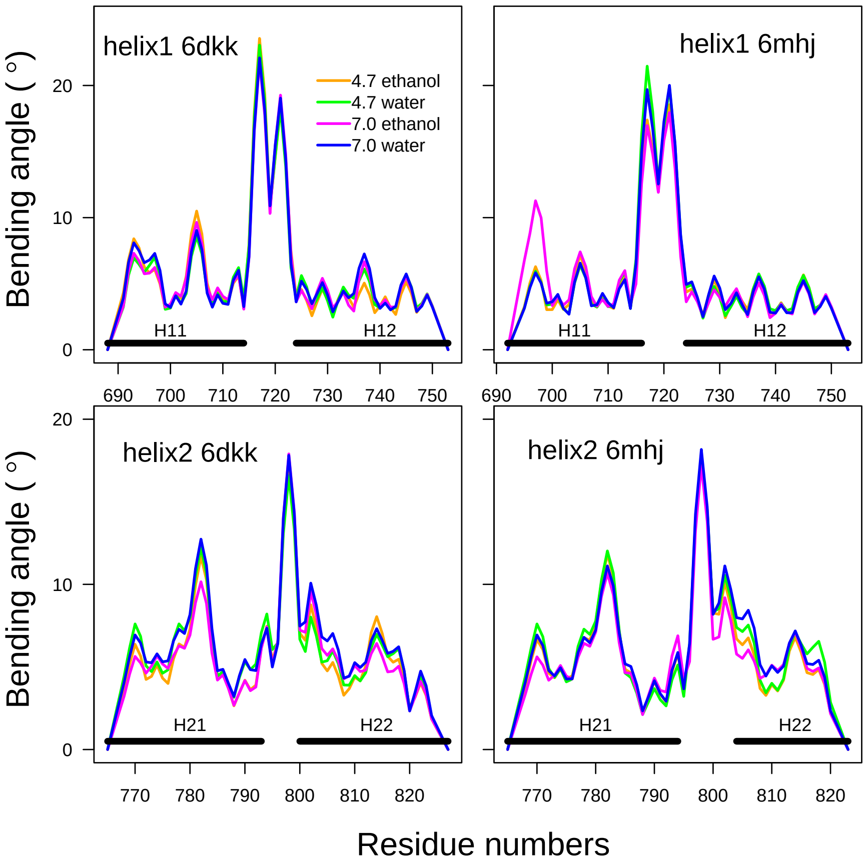

Figure S9: Bending angles of the helices 1 and 2. The analysis was performed using the package Bendix [2] on the second replicate of trajectories.

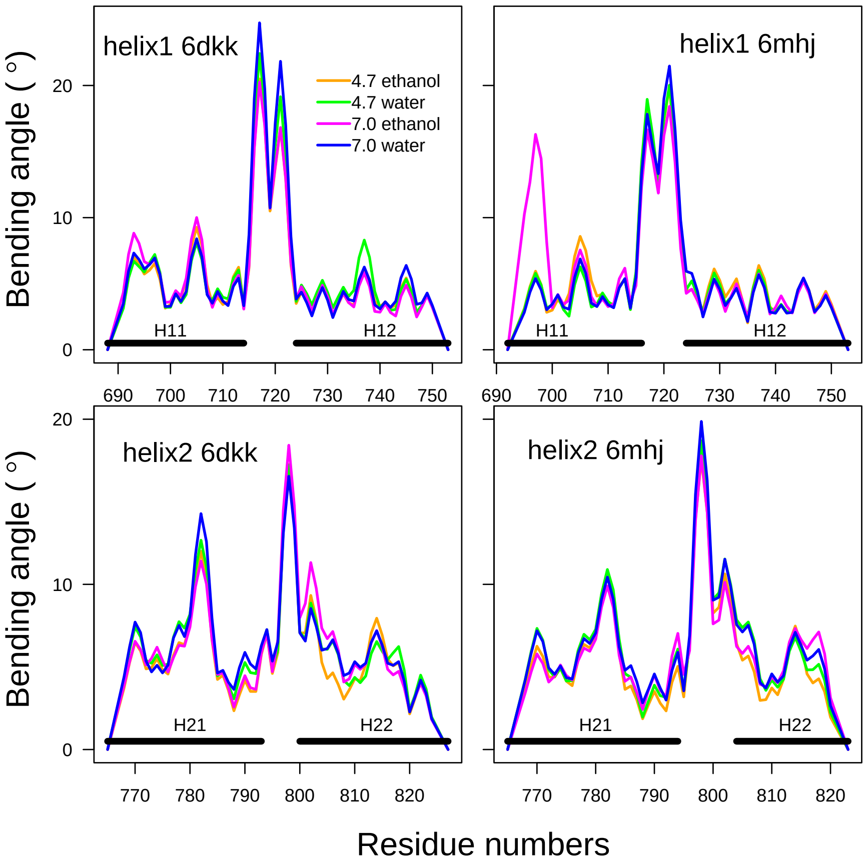

Figure S10: Bending angles of the helices 1 and 2. The analysis was performed using the package Bendix [2] on the third replicate of trajectories.

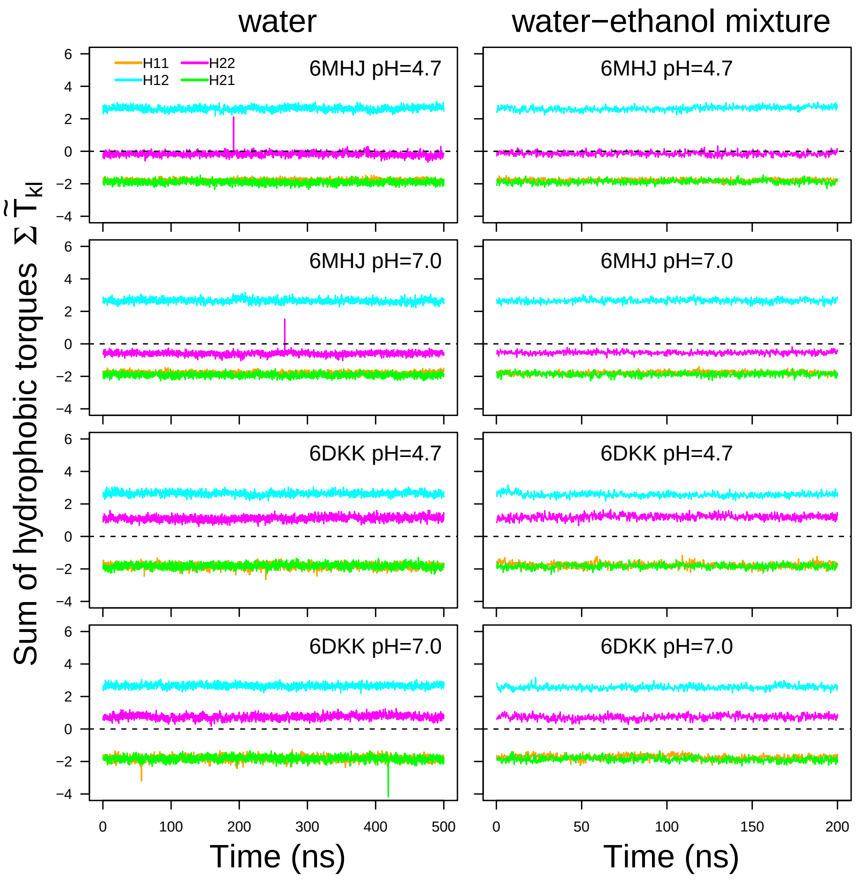

Figure S11: Torque values averaged along the residues 695-713 (H11: orange), 730-748 (H12: cyan), 766-784 (H21: green) and 801-819 (H22: magenta) and plotted along the time for the second replicate of trajectories. H12 and H21 are interacting through a coiled-coil motif, as well as H11 and H22. For pH 7, these values were, respectively, calculated on the following sets of hydrophobic residues: I695, A698, L699, W706, V709, I713 for H11, M732, A735, L736, A740, A742, A745, I746, I747 for H12, I766, L769, L773, I777, A780, M781, I782, I784 for H21, I801, P802, G804, V805, L808, F811, A813, L815, A818, L819 for H22. For pH 4.7, the protonated residue E809 was added to the list of H22. Note that the curves of H11 and H21 superimpose each other.

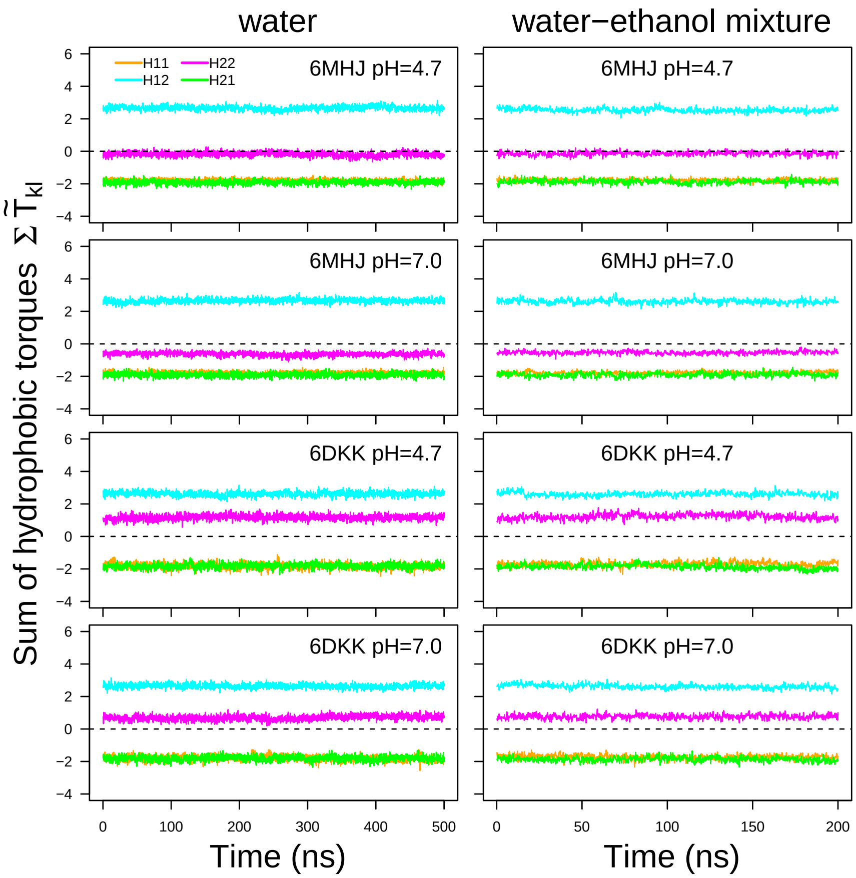

Figure S12: Torque values averaged along the residues 695-713 (H11: orange), 730-748 (H12: cyan), 766-784 (H21: green) and 801-819 (H22: magenta) and plotted along the time for the third replicate of trajectories. H12 and H21 are interacting through a coiled-coil motif, as well as H11 and H22. For pH 7, these values were, respectively, calculated on the following sets of hydrophobic residues: I695, A698, L699, W706, V709, I713 for H11, M732, A735, L736, A740, A742, A745, I746, I747 for H12, I766, L769, L773, I777, A780, M781, I782, I784 for H21, I801, P802, G804, V805, L808, F811, A813, L815, A818, L819 for H22. For pH 4.7, the protonated residue E809 was added to the list of H22. Note that the curves of H11 and H21 superimpose each other.

System	number of water molecules	number of ethanol molecules	counter-ions	total number of atoms	solvent box (Å,Å,Å)
6MHJ47W	30728	-	6 Cl-	97483	146.8, 82.7, 79.8
6MHJ47E	15544	15270	5 Cl-	189355	187.6, 103.6, 102.2
6MHJ70W	30734	-	2 Na+	97489	146.7, 82.7, 79.8
6MHJ70E	15542	15274	3 Na+	189375	187.6, 103.6, 102.3
6DKK47W	39954	-	3 Cl-	125155	127.2, 113.6, 86.3
6DKK47E	15195	15029	3 Cl-	186139	183.9, 104.1, 102.2
6DKK70W	39957	-	2 Na+	125157	127.1, 113.5, 86.2
6DKK70E	15201	15033	2 Na+	186186	184.0, 104.1, 102.2
water-ethanol box	16120	16120	-	193440	192.1, 104.1, 101.8

Table S1: Composition of the systems for molecular dynamics simulations. The box sizes are those observed at the end of equilibration.

PDB entry	Residues	RMSD (Å)	ω_0 (deg)	α (deg)	Rise (Å)
6MHJ	695-713/801-819	0.643	1.636	5.958	1.503
6MHJ	730-748/766-784	0.472	3.644	13.019	1.514
6DKK	695-713/801-819	0.59	1.742	6.465	1.497
6DKK	730-748/766-784	0.579	3.881	14.013	1.498

Table S2: Crick parameters determined on the residues ranges extracted from PDB entries 6MHJ and 6DKK, using the software CCCP (Coiled-coil Crick Parameterization) available at the Web server: `www.grigoryanlab.org/cccp` [3]. For 6DKK, the calculation was performed on the chain A. The RMSD value is calculated between the PDB structure and the ideal geometry determined using the measured Crick parameters. Some Crick parameters are given in the Table: Superhelical frequency (ω_0), pitch angle (α), rise per residue (Rise).

PDB entry	pH	helix	Number of residues	residue list
6MHJ	4.7	helix 1	23	692, 695, 698, 699, 706, 709, 713, ***714***, 717, 718, 719, 721, ***725***, 727, 728, 732, 735, 736, 740, 742, 745, 746, 747
6MHJ	4.7	helix 2	27	766, 769, 773, 777, 780, 781, 782, 784, 787, 788, ***793***, 796, 797, 800, 801, 802, ***804***, 805, 808, **809**, 811, 813, 815, 818, 819, 820, 823
6MHJ	7.0	helix 1	23	692, 695, 698, 699, 706, 709, 713, ***714***, 717, 718, 719, 721, ***725***, 727, 728, 732, 735, 736, 740, 742, 745, 746, 747
6MHJ	7.0	helix 2	26	766, 769, 773, 777, 780, 781, 782, 784, 787, 788, ***793***, 796, 797, 800, 801, 802, ***804***, 805, 808, 811, 813, 815, 818, 819, 820, 823
6DKK	4.7	helix 1	25	689, 690, 692, 695, 698, 699, 706, 709, 713, ***714***, 717, 718, 719, 721, ***725***, 727, 728, 732, 735, 736, 740, 742, 745, 746, 747
6DKK	4.7	helix 2	27	766, 769, 773, 777, 780, 781, 782, 784, 787, 788, ***793***, 796, 797, ***800***, 801, 802, 804, 805, 808, **809**, 811, 813, 815, 818, 819, 820, 823
6DKK	7.0	helix 1	25	689, 690, 692, 695, 698, 699, 706, 709, 713, ***714***, 717, 718, 719, 721, ***725***, 727, 728, 732, 735, 736, 740, 742, 745, 746, 747
6DKK	7.0	helix 2	26	766, 769, 773, 777, 780, 781, 782, 784, 787, 788, ***793***, 796, 797, ***800***, 801, 802, 804, 805, 808, 811, 813, 815, 818, 819, 820, 823

Table S3: Hydrophobic residues for helices 1 and 2 depending on the PDB structures and pH values. The residue 809, which is protonated at pH 4.7, is written in bold. The residues defining the direction of the resulting hydrophobic strip on the helices are written in bold italic, as described in Sec. III.C in the main text.

References

[1] William Humphrey, Andrew Dalke, and Klaus Schulten. VMD – Visual Molecular Dynamics. *Journal of Molecular Graphics*, 14:33–38, 1996.

[2] A. C. Dahl, M. Chavent, and M. S. Sansom. Bendix: intuitive helix geometry analysis and abstraction. *Bioinformatics*, 28:2193–2194, 2012.

[3] G. Grigoryan and W. F. Degrado. Probing designability via a generalized model of helical bundle geometry. *J Mol Biol*, 405(4):1079–1100, 2011.
